# The Heterooligomerization of Human Small Heat Shock Proteins Is Controlled by Conserved Motif Located in the N-Terminal Domain

**DOI:** 10.3390/ijms21124248

**Published:** 2020-06-15

**Authors:** Vladislav M. Shatov, Sergei V. Strelkov, Nikolai B. Gusev

**Affiliations:** 1 Department of Biochemistry, School of Biology, Moscow State University, Moscow 119991, Russian; shatovm@inbox.ru; 2 Laboratory for Biocrystallography, Department of Pharmaceutical and Pharmacological Sciences, KU Leuven, 3000 Leuven, Belgium; sergei.strelkov@kuleuven.be

**Keywords:** small heat shock proteins, HspB1, HspB5, HspB6, HspB8, heterooligomerization

## Abstract

Ubiquitously expressed human small heat shock proteins (sHsps) HspB1, HspB5, HspB6 and HspB8 contain a conserved motif (S/G)RLFD in their N-terminal domain. For each of them, we prepared mutants with a replacement of the conserved R by A (R/A mutants) and a complete deletion of the pentapeptide (Δ mutants) and analyzed their heterooligomerization with other wild-type (WT) human sHsps. We found that WT HspB1 and HspB5 formed heterooligomers with HspB6 only upon heating. In contrast, both HspB1 mutants interacted with WT HspB6 even at low temperature. HspB1/HspB6 heterooligomers revealed a broad size distribution with equimolar ratio suggestive of heterodimers as building blocks, while HspB5/HspB6 heterooligomers had an approximate 2:1 ratio. In contrast, R/A or Δ mutants of HspB6, when mixed with either HspB1 or HspB5, resulted in heterooligomers with a highly variable molar ratio and a decreased HspB6 incorporation. No heterooligomerization of HspB8 or its mutants with either HspB1 or HspB5 could be detected. Finally, R/A or Δ mutations had no effect on heterooligomerization of HspB1 and HspB5 as analyzed by ion exchange chromatography. We conclude that the conserved N-terminal motif plays an important role in heterooligomer formation, as especially pronounced in HspB6 lacking the C-terminal IXI motif.

## 1. Introduction

Small heat shock proteins (sHsp) form a large family of ubiquitously expressed proteins playing an important role in proteostasis [1,2,3]. Human genomes contains ten genes encoding sHsps [4,5] and four members (HspB1, HspB5, HspB6 and HspB8) of this large family are expressed in practically all human tissues [6,7]. In certain tissues (for instance, in skeletal muscles and heart), some of these proteins are simultaneously expressed at comparably high level, reaching up to 3–5% of the total soluble proteins [8]. 

Small heat shock proteins tend to form oligomers of various size [9,10]. In line with a considerable conservation of primary structure across different sHsps [11], they are capable of forming heterooligomeric complexes [12,13,14] which are structurally and functionally distinct from the corresponding homooligomers. The formation of heterooligomeric complexes formed by HspB1 and HspB5 [14,15], HspB5 and HspB6 [14,16,17,18], HspB8 and other sHsps [19,20] has been reported in the literature. The interaction of HspB1 and HspB6 and their ability to form heterooligomeric complexes has been analyzed especially rigorously [21,22,23,24]. Special attempts were undertaken in order to establish the formation of heterooligomeric complexes among all four ubiquitously expressed sHsps (HspB1, HspB5, HspB6, HspB8) [19,25,26]. 

It is well-accepted that all parts of sHsps, i.e., N-terminal (NTD), α-crystallin (ACD) and C-terminal domains (CTD) can play significant role in the formation of heterooligomeric complexes [18]. The α-crystallin domain of sHsps is highly conserved [11] and therefore cannot determine specificity of heterooligomerization. At the same time, it is known that the interaction between sHsps is highly specific and depends on the nature of protein partners [13,25,26]. Human sHsps differ in the presence or absence of conserved IX(I/V) tripeptide located in the C-terminal domain possessing high mobility and involved in formation of intra- and inter-subunit contacts in large oligomeric complexes [27]. In addition, sHsps differ in the size and composition of their N-terminal domains playing important role in stabilization of large homooligomers [9,28,29,30,31]. Therefore, the specificity of heterooligomerization seems to be predominantly dependent on the structure and composition of variable C- and N-terminal domains of sHsps.

**Figure 1:**
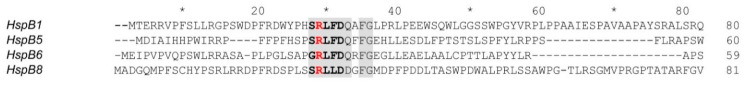
Alignment of the primary structure of the N-terminal domains (NTD) of four human small heat shock proteins (sHsp). The conserved (S/G)RLFD(D/Q)xFG motif is marked grey, the conserved pentapeptide is depicted in bold letters, the conserved Arg residue is marked red. The following sequences from Uniprot were used: P04792 (HspB1), P02511 (HspB5), O14558 (HspB6), Q9UJY1 (HspB8). Multiple alignment was generated by Tcoffee.

Ubiquitously expressed human sHsps (HspB1, HspB5, HspB6 and HspB8) differ in the size and structure of their N-terminal domains. The latter play an important role in chaperone-like activity [30,32,33], in formation of homooligomers [9,10,29,31,32] and in formation of heterooligomeric complexes [18,23,24]. Although the length and the primary structure of the N-terminal domain of sHsps is highly variable [11], all these proteins contain a conserved (S/G)RLFD(D/Q)xFG motif located at the beginning of this domain (Figure 1). In order to analyze the role of this motif in the process of heterooligomerization, we replaced highly conserved Arg in this sequence with Ala (R/A mutants) or completely deleted the first five residues of this motif (Δ mutants) and compared the pairwise heterooligomerization of the wild-type (WT) proteins and their R/A and Δ mutants by means of size exclusion or ion-exchange chromatography.

## 2. Results

### 2.1. Characterization of Analyzed Proteins 

**Figure 2:**
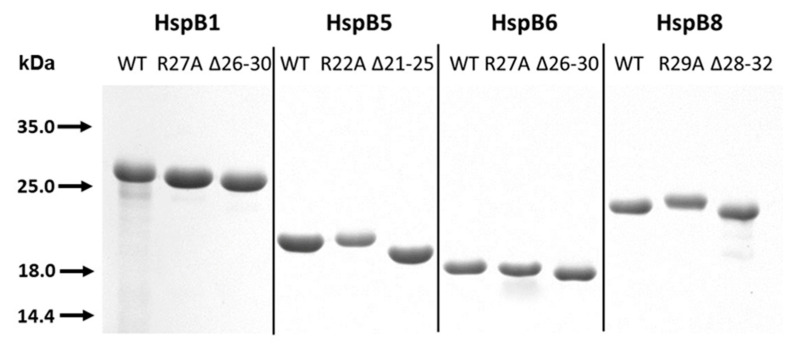
Sodium dodecyl sulfate (SDS) gel electrophoresis of the final preparation of the wild-type (WT) proteins and their mutants. About 1.5 µg of proteins were loaded on each track.

The method of expression and purification provided highly purified samples of the WT sHsps and their R/A and Δ mutants (Figure 2). Point mutation R/A had no noticeable effect on electrophoretic mobility, whereas the deletion of pentapeptide was accompanied by a small increase of electrophoretic mobility of all proteins on sodium dodecyl sulfate (SDS) gel electrophoresis (Figure 2).

### 2.2. Effect of R/A and Δ Mutations on the Interaction of HspB1 and HspB6

**Figure 3:**
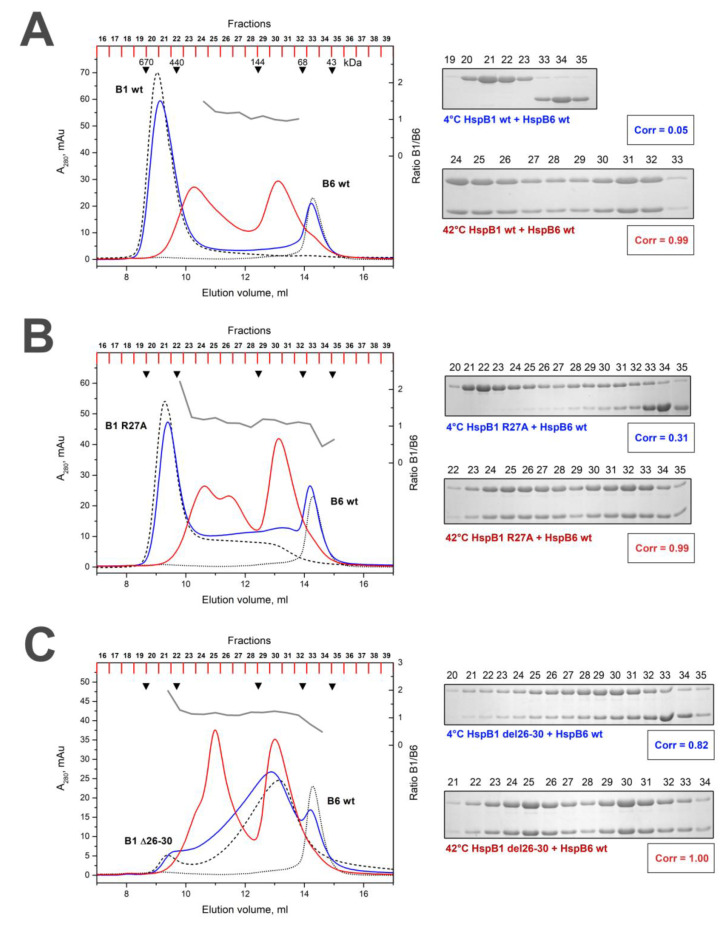
Size-exclusion chromatography (SEC) of the WT HspB1 (**A**), and its R/A (**B**) and Δ (**C**) mutants and their mixtures with the WT HspB6. Elution profiles of isolated HspB6 are shown as a black dotted line; that of isolated HspB1 or its mutants by black dashed line. The elution profile of the mixture of HspB1 species and HspB6 preincubated at low temperature is shown as a solid blue line and that of the corresponding mixture preincubated at elevated temperature is shown as solid red line. The molar ratio HspB1/HspB6 (grey solid line) in the corresponding fractions obtained after chromatography of preheated sample is indicated on the right ordinate. The protein composition of the fractions obtained after chromatography of HspB1/HspB6 mixture preincubated at low (labeled blue) or high (labeled red) temperature are presented on inserts. Correlation coefficient (Corr) is indicated under each electropherogram. The numbers above tracks correspond to the fractions number. Apparent molecular weights are indicated by arrows.

The WT HspB1 forms large oligomers with an apparent molecular mass of 530-600 kDa [30,34]. As reported earlier [35], the R/A mutation resulted in the partial dissociation of large oligomers of HspB1 and deletion of the conserved pentapeptide resulted in complete dissociation of large HspB1 oligomers and accumulation of small oligomers with apparent molecular weight of 90 kDa (Figure 3). The R/A or Δ mutations had no effect on the quaternary structure of HspB6, and both the WT protein and its R/A and Δ mutants formed only small oligomers with an apparent molecular weight of 58–60 kDa on the size exclusion chromatography (SEC).

In line with previous observations [21,22], mixing equimolar amounts of WT HspB1 and HspB6 at 42 °C resulted in the formation of a broad spectrum of heterooligomeric complexes in a SEC experiment, with apparent molecular weight peaking at about 100 and 300 kDa (Figure 3). The stoichiometry of HspB1/HspB6 was close to equimolar in all chromatographic fractions. Towards further quantification, here we have introduced a correlation coefficient based on the presence of either partner across the chromatographic fractions (see Methods). Such a correlation coefficient is a convenient integral measure which reflects the propensity of a given pair of sHsps to form heterocomplexes. For heated WT HspB1 and HspB6 samples, the correlation coefficient was close to 1, indicating a strong preference towards heterooligomerization consistent with previous reports [24]. In contrast, unheated mix of HspB1 and HspB6 resulted in a correlation coefficient close to 0. This reflects the fact that the two proteins do not interact and elute in very different parts of the chromatogram, namely as large HspB1 homooligomers and much smaller HspB6 homodimers (Figure 3).

When the mixture of R/A mutant of HspB1 and the WT HspB6 preincubated at low temperature was loaded on the column, the HspB1 mutant was detected in practically all fractions of the elution profile, including the fractions 34–35 containing proteins with an apparent molecular weight of 58–60 kDa (Figure 3B). The molar ratio HspB1/HspB6 in these complexes varied in the range of 5:1 to 1:1 (Table 1). This means that even at a low temperature, the R/A mutant of HspB1 can interact and form heterooligomers with the WT HspB6 with variable stoichiometry. At a high temperature, this mutant formed three different heterooligomeric complexes with the WT HspB6 with apparent molecular weights of 100, 210 and 300 kDa. Stoichiometry HspB1/HspB6 in all these complexes was close to equimolar. 

After the mixture of Δ mutant of HspB1 and WT HspB6 preincubated at low temperature had been loaded on the column, we detected a decrease in the peak corresponding to isolated HspB6 (fractions 33–35) and appearance of HspB6 in fractions 21–27 where it was eluted together with Δ mutant of HspB1 (Figure 3C). This was apparent in a correlation coefficient of 0.82 between the elution profiles for both components. This means that even at low temperature Δ mutant of HspB1 forms heterooligomeric complexes with the WT HspB6 with variable stoichiometry (Table 1). When heated mixture of Δ mutant of HspB1 and WT HspB6 was loaded on the column, we detected two peaks of heterooligomers with apparent molecular weight of 100 and 260 kDa, where stoichiometry HspB1/HspB6 was constant and close to 1:1 (Figure 3C). Interestingly, the WT HspB6 was able to promote re-association of Δ mutant of HspB1 and formation of heterooligomeric complexes with high molecular weight (fractions 23–26 on Figure 3C).

**Figure 4:**
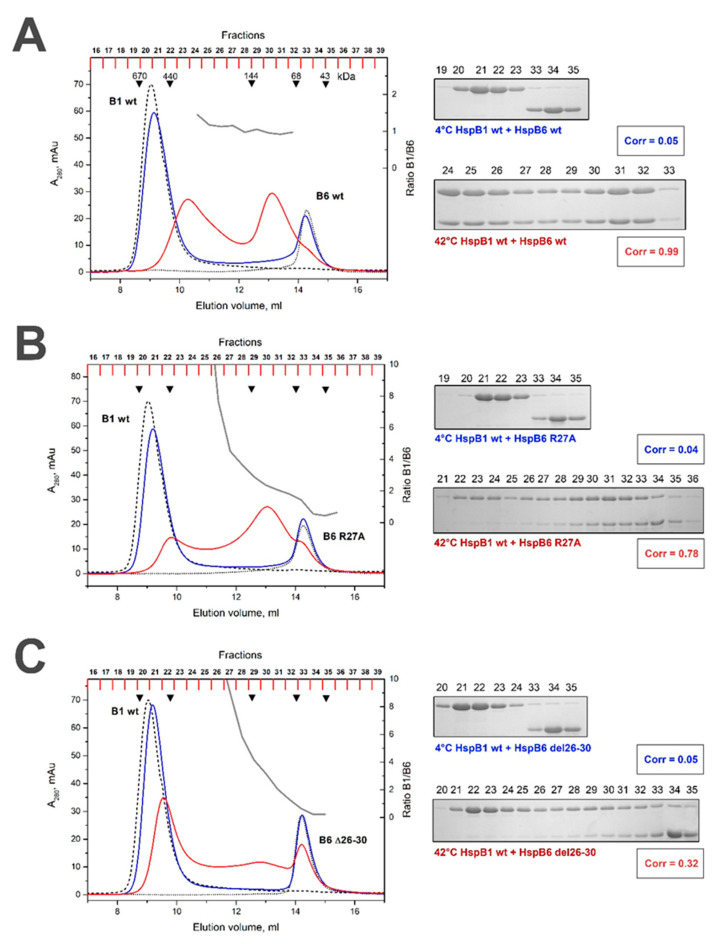
Size-exclusion chromatography of the WT HspB6 (**A**), its R/A (**B**) and Δ (**C**) mutants and their mixtures with the WT HspB1. The elution profiles of isolated HspB6 or its mutants are shown as a black dotted line, that of isolated WT HspB1 by a black dashed line. The elution profile of the mixture of HspB6 species and WT HspB1 preincubated at low temperature is shown as a solid blue line and that of the corresponding mixture preincubated at elevated temperature is shown as a solid red line. The molar ratio HspB1/HspB6 (grey solid line) in the corresponding fractions obtained after chromatography of preheated sample is indicated on the right ordinate. The protein composition of the fractions obtained after the chromatography of HspB1/HspB6 mixture preincubated at low (labeled blue) or high (labeled red) temperature are presented on the inserts. The correlation coefficient (Corr) is indicated under each electropherogram. The numbers above tracks correspond to the fractions number. Apparent molecular weights are indicated by arrows.

At a low temperature, the R/A mutant of HspB6 was unable to form heterooligomeric complexes with the WT HspB1 (Figure 4B). Next, a mixture of R/A mutant of HspB6 and the WT HspB1 was heated to 42 °C prior to chromatography. The WT HspB1 was detected in practically all fractions and the stoichiometry HspB1/HspB6 varied between 10:1 and 1:1 (Table 1). The only one pronounced peak with apparent molecular weight of ~100 kDa (fractions 29–31 in Figure 4B) contained a constant ratio of HspB1 and R/A mutant of HspB6 that was close to 2:1. At low temperature Δ mutant of HspB6 was also unable to form heterooligomeric complexes with the WT HspB1 (Figure 4C). At high temperatures, Δ mutant of HspB6 induced partial dissociation of large oligomers of HspB1 and therefore HspB1 was distributed over the whole elution profile (Figure 4C) without being able to form any complexes with fixed stoichiometry with the Δ mutant of HspB6 (Table 1). 

### 2.3. Effect of R/A and Δ Mutations on the Interaction of HspB5 and HspB6

**Figure 5:**
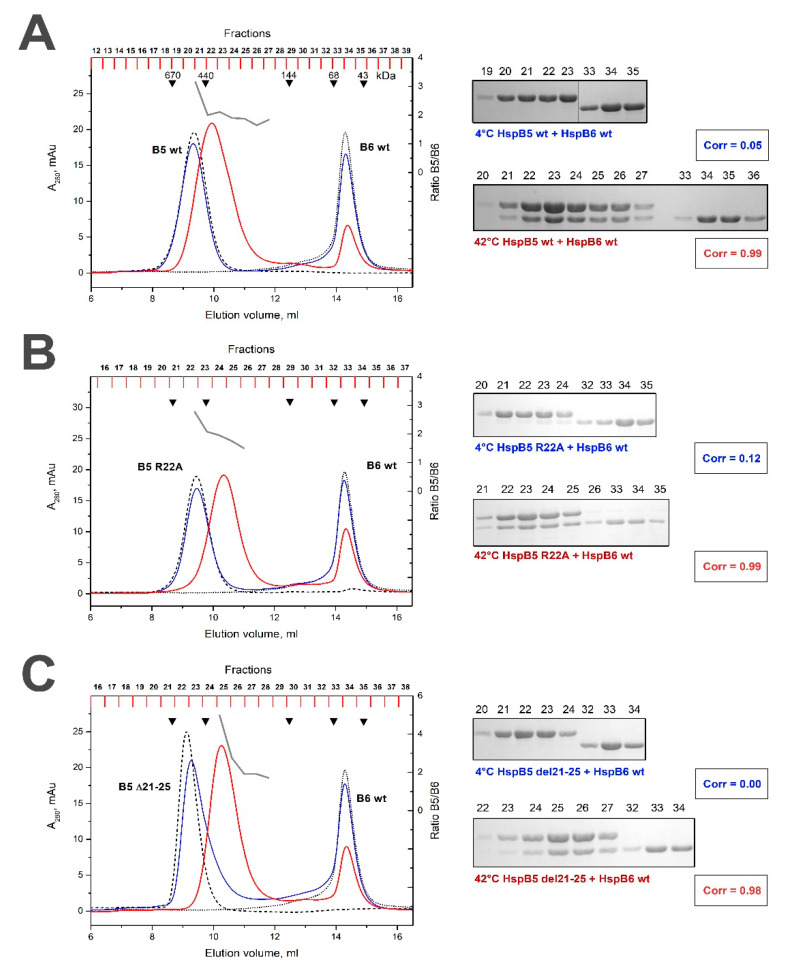
Size-exclusion chromatography of the WT HspB5 (**A**), its R/A (**B**) and Δ (**C**) mutants and their mixtures with the WT HspB6. The elution profiles of isolated HspB6 are shown as a black dotted line, that of isolated WT HspB5 or its mutants by a black dashed line. The elution profile of the mixture of HspB5 species and WT HspB6 preincubated at low temperature is shown as a solid blue line and that of the corresponding mixture preincubated at elevated temperature is shown as a solid red line. The molar ratio HspB5/HspB6 (grey solid line) in the corresponding fractions obtained after chromatography of preheated sample is indicated on the right ordinate. The protein composition of the fractions obtained after the chromatography of HspB5/HspB6 mixture preincubated at low (labeled blue) or high (labeled red) temperature are presented on inserts. The correlation coefficient (Corr) is indicated under each electropherogram. The numbers above tracks correspond to the fractions number. Apparent molecular weights are indicated by arrows.

The WT HspB5 and its R/A or Δ mutants formed large stable oligomers with apparent molecular weights of 510–550 kDa [35]. At low temperature the WT HspB5 was unable to form heterooligomeric complexes with the WT HspB6 and each protein was eluted in separate peak with apparent molecular weight of 540–550 and 58–60 kDa (Figure 5A). After heating, the WT HspB5 and the WT HspB6 formed one type of heterooligomers with an apparent molecular weight of 400 kDa (Figure 5A). The stoichiometry of HspB5/HspB6 in this complex was close to 2:1 (Table 1) and about half of HspB6 remained unbound and was not included in heterooligomers. The R/A mutant of HspB5 formed heterooligomers with the WT HspB6 only at elevated temperature. These complexes had apparent molecular weight of 350 kDa and the stoichiometry HspB5/HspB6 varied in the range of 3:1 to 2:1 (Table 1). Under these conditions, a larger portion of HspB6 remained free and was not included in heterooligomeric complex. Similar results were obtained with Δ mutant of HspB5. Indeed, heterooligomeric complexes were formed only at elevated temperature, had an apparent molecular weight of ~350 kDa and stoichiometry HspB5/HspB6 varying in the range 5:1 and 2:1 (Figure 5C, Table 1).

**Figure 6:**
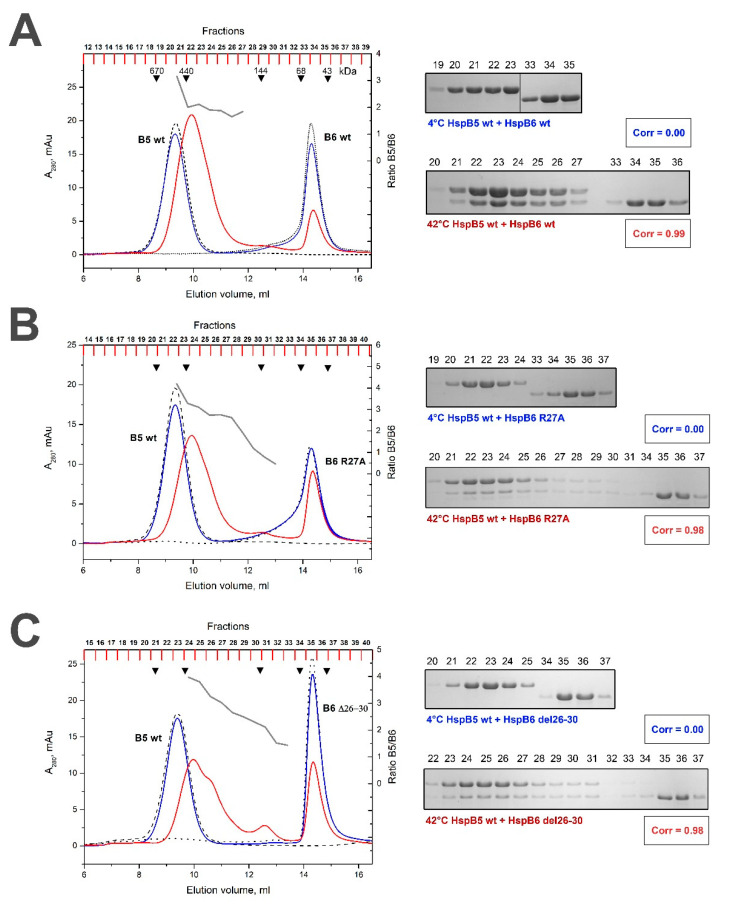
Size-exclusion chromatography of the WT HspB6 (**A**), its R/A (**B**) and Δ (**C**) mutants and their mixtures with the WT HspB5. The elution profiles of isolated HspB6 or its mutants are shown as a black dotted line, that of isolated WT HspB5 by a black dashed line. The elution profile of the mixture of HspB6 species and WT HspB5 preincubated at low temperature is shown as a solid blue line and that of the corresponding mixture preincubated at elevated temperature is shown as a solid red line. The molar ratio HspB5/HspB6 (grey solid line) in the corresponding fractions obtained after the chromatography of preheated sample is indicated on the right ordinate. Protein compositions of fractions obtained after chromatography of HspB5/HspB6 mixture preincubated at low (labeled blue) or high (labeled red) temperature are presented on inserts. The correlation coefficient (Corr) is indicated under each electropherogram. The numbers above tracks correspond to the fractions number. Apparent molecular weights are indicated by arrows.

At low temperature neither R/A nor Δ mutants of HspB6 were able to form heterooligomeric complexes with the WT HspB5 (Figure 6B,C). At high temperature these mutants of HspB6 formed one type of heterooligomers with HspB5 with apparent molecular weight of 400 kDa. The stoichiometry HspB5/HspB6 in this heterogeneous complex was variable (Table 1) and significant portion of HspB6 mutants remained free and were not included in heterooligomeric complex (Figure 6B,C).

### 2.4. Lack of Interaction of HspB8 and Its Mutants with HspB1 and HspB5

**Figure 7:**
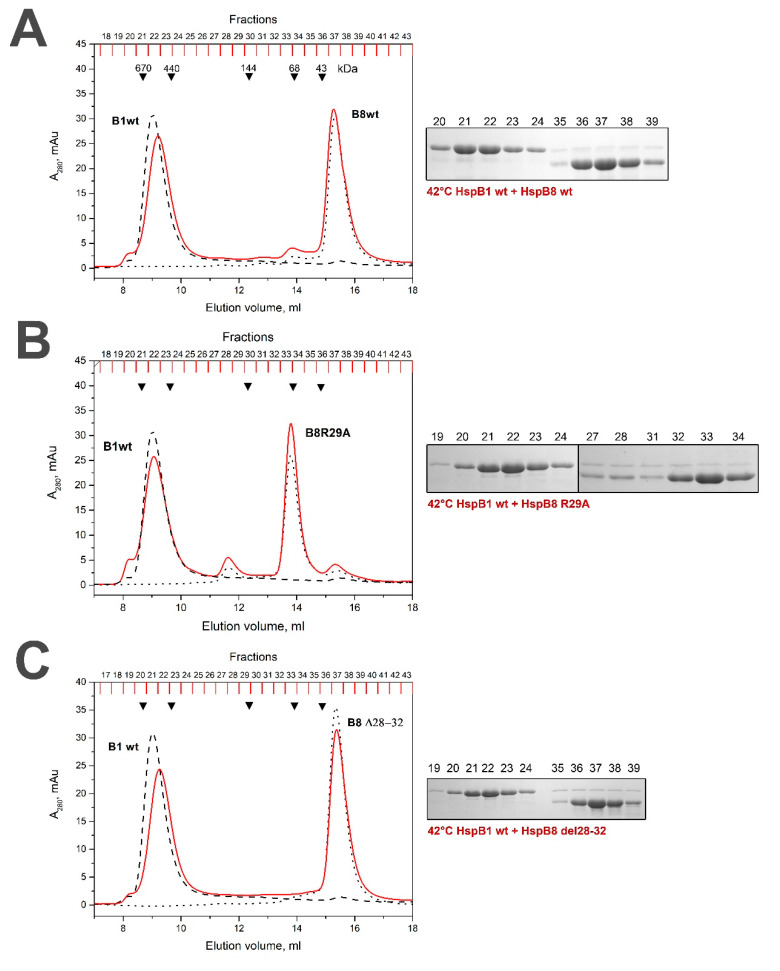
Size exclusion chromatography of the WT HspB1 and the WT HspB8 (**A**) or its R/A (**B**) or Δ (**C**) mutants. The elution profile of HspB8 and its mutants is shown as a black dotted line and that of isolated HspB1 as a black dashed line. The elution profile of the mixture of HspB1 and HspB8 species preincubated at elevated temperature is shown as a solid red line. The protein compositions of fractions obtained after chromatography of HspB1/HspB8 mixture preincubated at high temperature are presented on inserts. The numbers above tracks correspond to the fractions number. Apparent molecular weights are indicated by arrows.

When the mixture of the WT HspB1 and the WT HspB8 preincubated at high temperature was loaded on the Superdex 200 column, we detected two separate peaks with apparent molecular weights of 590 and 35 kDa, each of which contained isolated HspB1 and HspB8, respectively (Figure 7A). Similar results were obtained in the case of the mixture containing R/A mutant of HspB8 and the WT HspB1 (Figure 7B), except for the fact that, as expected, the apparent molecular weight of the R/A mutant of HspB8 was larger than that of the WT protein and was close to 65 kDa [35]. The Δ mutant of HspB8 was also unable to interact with the WT HspB1 and two proteins again were eluted in separate peaks (Figure 7C). Qualitatively similar results were obtained with the mixture of the WT HspB8 and the WT HspB5 or its R/A or Δ mutants. Thus, even at elevated temperature HspB8 was unable to interact with HspB1 or HspB5.

### 2.5. Formation of Heterooligomeric Complexes between HspB1 and HspB5

**Figure 8:**
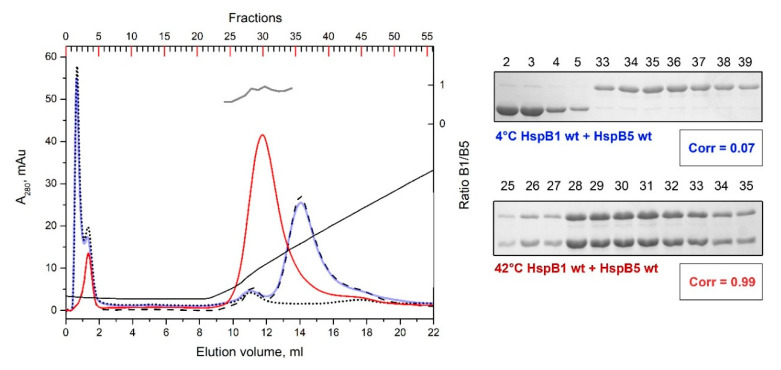
Ion-exchange chromatography of the WT HspB1 and HspB5. The elution profile of isolated HspB1 and HspB5 are shown as black dashed and dotted lines, respectively. The elution profile of the mixture of HspB1 and HspB5 preincubated at low temperature is shown as a solid blue line and that of the corresponding mixture preincubated at elevated temperature is shown as a solid red line. The molar ratio HspB1/HspB5 (grey solid line) in corresponding fractions obtained after chromatography of preheated sample is indicated on the right ordinate. Conductivity is shown as a solid black line. The protein compositions of fractions obtained after chromatography of HspB1/HspB5 mixture preincubated at low (labeled blue) or high (labeled red) temperature are presented on inserts. Correlation coefficient (Corr) is indicated under each electropherogram. The numbers above tracks correspond to the fractions number.

Both HspB1 and HspB5 form similar size large homooligomers, and therefore the formation of heterooligomeric complexes of these proteins cannot be followed by means of size-exclusion chromatography. At the same time, these proteins have different isoelectric points, and therefore can be separated by means of ion-exchange chromatography. At pH 6.8, isolated HspB5 with a theoretical isoelectric point 6.76 was not retarded on the DEAE HiTrap column, whereas HspB1 with a theoretical isoelectric point 5.98 was tightly bound to the column and was eluted only by salt gradient (Figure 8). When the mixture of the WT HspB1 and HspB5 preincubated at low temperature was loaded on the DEAE HiTrap column, HspB5 was not retarded on the column and was eluted in the flow through, whereas HspB1 was retarded on the column and eluted by salt gradient in a position corresponding to the isolated protein (Figure 8). Only one peak was detected on the elution profile of ion exchange chromatography when a preheated mixture of HspB1 and HspB5 was loaded on the DEAE HiTrap column (Figure 8). This peak was less tightly bound to the column than isolated HspB1 and contained equimolar mixture of HspB1 and HspB5 (Figure 8). We have run similar experiments with different mixtures containing R/A or Δ mutants of HspB1 and/or HspB5 and have not detected any differences in behavior of the WT proteins and their mutants. Thus, mutations in the N-terminal domain negligibly affect interaction of HspB1 and HspB5.

## 3. Discussion

The heterooligomerization of various human sHsps has been frequently reported [14,25,26], although the physiological implications of this process are still poorly understood. The experiments reported here indicate an important involvement of the conserved pentapeptide within the N-terminal motif of selected human sHsps on their heterooligomerization properties.

Although the whole peptide (S/G)RLFD in the N-terminal domain is conserved, the single positively charged Arg residue is especially presented in practically all vertebrate sHsp homologues (HspB1, HspB2, HspB4, HspB5, HspB6 and HspB8) [30]. Point mutation R/A decreases the positive charge in the N-terminal part of HspB1 and results in a partial dissociation of large HspB1 oligomers (Figure 3). In this respect, the R/A replacement is similar to that induced by Ser15 phosphorylation leading to decrease of positive and increase of negative charge of the N-terminal domain and provoking dissociation of HspB1 [34,36]. We can speculate that the decrease of the positive (or increase of negative) charge of the N-terminal domain induces dissociation of large HspB1 oligomers. In line with previous observations [23,24], the R/A mutant of HspB1 formed heterooligomers with the WT HspB6 even at low temperature that is unusual for the WT proteins (Table 1). This can be due to more effective heterooligomerization of already partially dissociated species of HspB1. At high temperatures, the R/A mutant of HspB1 formed different heterooligomers with HspB6, with apparent molecular weights of 100, 210 and 300 kDa, each of which contained equimolar quantities of both sHsps (Figure 3B, Table 1).

The deletion of the pentapeptide SRLFD induced a dramatic destabilization of HspB1 homooligomers and the Δ mutant of HspB1 was presented in the form of heterogeneous small oligomers (Figure 3C). Even at low temperature, these small oligomers of the HspB1 Δ mutant formed a heterogeneous mixture of heterooligomers with the WT HspB6, with variable stoichiometry and correlation coefficient close to 0.82 (Table 1). At elevated temperatures, the Δ mutant of HspB1 formed two types of heterooligomeric complexes with HspB6, with apparent molecular weights of 100 and 260 kDa with fixed stoichiometry close to 1:1, and practically indistinguishable from those formed by the WT HspB1 (Figure 3C, Table 1). It is remarkable that under these conditions HspB6 was able to reconstitute large oligomers that were completely absent in the case of the isolated Δ mutant of HspB1. 

Neither the R/A mutation [35] nor the deletion of the conserved peptide affected the quaternary structure of HspB6, and both the WT protein and its mutants were eluted on size exclusion chromatography in the peak, with an apparent molecular weight of 58–60 kDa (Figure 4). These data agree with the earlier published results of Heirbaut et al. [24,33]. Heterooligomers formed by the R/A mutant of HspB6 and the WT HspB1 at high temperatures were very heterogeneous. Stoichiometry HspB1/HspB6 in these complexes was variable, and, in the most prominent heterocomplex, with an apparent molecular weight of 100 kDa, was close to 2:1 (Figure 4B, Table 1). After preincubation at elevated temperatures, the Δ mutant of HspB6 induced partial dissociation of large oligomers of HspB1 and different oligomers species of HspB1 were distributed over the whole elution profile without forming a separate peak of heterooligomer with fixed stoichiometry (Figure 4C, Table 1). Thus, mutations in the N-terminal part of HspB6 impair its interaction with HspB1. 

Both the R/A and Δ mutations had only a marginal effect on the quaternary structure of HspB5 (Figure 5). The heterooligomeric complexes of the WT HspB5 and WT HspB6 were formed only at high temperature and had an apparent molecular weight of 400 kDa and fixed stoichiometry HspB5/HspB6 close to 2:1 (Table 1). The R/A mutant of HspB5 also interacted with HspB6 only at elevated temperature and formed heterooligomers with an apparent molecular weight of 350 kDa and stoichiometry HspB5/HspB6 varying in the range of 3:1 to 2:1 (Figure 5B). In this case, a larger portion of HspB6 remained free and was not involved in complex formation. Similar results were obtained with the Δ mutant of HspB5 (Figure 5C). Thus, mutations in the N-terminal of HspB5 moderately affect its interaction with the WT HspB6. The stoichiometry HspB5/HspB6 in complexes formed by HspB5 mutants was variable and larger than in the case of the WT HspB5, thus leaving larger portions of HspB6 free.

The R/A mutant of HspB6 formed heterooligomers with the WT HspB5 only at elevated temperatures (Figure 6B). These complexes had a molecular weight of 350 kDa and variable stoichiometry HspB5/HspB6 (Table 1). A rather large portion of HspB6 remained free and was eluted in a separate peak (Figure 6B). Similar results were obtained with the Δ mutant of HspB6. In this case, three poorly separated peaks of heterooligomers were detected in the elution profile (Figure 6C). The stoichiometry HspB5/HspB6 in these complexes varied in the range of 4 to 1 and again a large portion of HspB6 and was not involved in formation of heterooligomeric complexes (Table 1).

Interestingly, in stark contrast with the exact 1:1 molar ratio of HspB1 and HspB6 in the heterooligomers which can be explained by the preferred formation of heterodimers, the heterooligomers formed by the WT HspB5 and HspB6 reveal a preferred 2:1 molar ratio (Figure 5). Several other sHsps were previously shown to heterooligomerize in ratios different from 1:1 (HspB2/HspB3 [37], HspB4/HspB5 [38]) Here, N-terminal mutations in HspB6, but not in HspB5, have a negative effect on the incorporation of HspB6 into the heterooligomers, while the ratio of HspB5 over HspB6 increases up to 3:1 and more (Figure 6, Table 1).

Our attempts to detect the interaction of the WT HspB8 or its N-terminal mutants with the WT HspB1 were unsuccessful (Figure 7). Neither at low (data not presented) nor at high temperature did the WT HspB8 or its N-terminal mutants form heterooligomers with the WT HspB1, and each protein was eluted in separate peaks. Similar results were obtained with HspB5 and HspB8 (data not presented). These data agree with our earlier published data obtained by means of chemical crosslinking [25] and disagree with results obtained by means of fluorescence resonance energy transfer (FRET), yeast two hybrid assay or immunoprecipitation [19,20].

The interaction of HspB1 and HspB5 using ion-exchange chromatography (Figure 8) revealed heterooligomeric complexes which were formed only at elevated temperatures. The stoichiometry of these complexes was close to 1:1. Neither the R/A nor Δ mutations of HspB1 or HspB5 had any effect on heterooligomer formation (data not shown). This means that mutations in the N-terminal domain did not affect heterooligomerization of HspB1 and HspB5.

**Figure 9:**
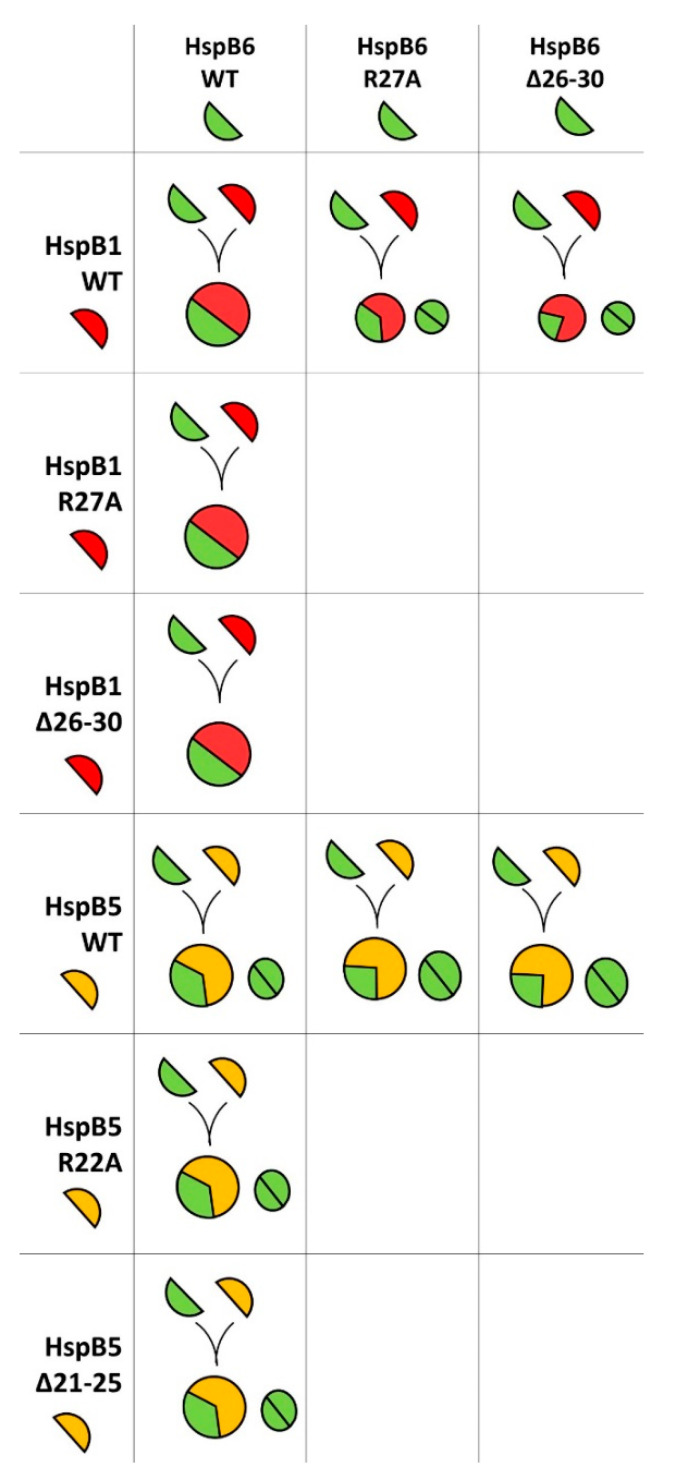
Hypothetical scheme of heterooligomerization of HspB1, HspB5 and HspB6 and their N-terminal mutants. HspB1, HspB5 and HspB6 monomers are represented as red, yellow and green half circles, respectively. Full circles represent homo- or heterooligomers and the colored area corresponds to the portion of the corresponding protein in heterooligomer. The interaction of HspB1 and HspB6. The WT HspB1 and HspB6 form heterooligomeric complexes containing similar 1:1 ratio of two proteins. Mutations of HspB1 NTD weakly affect its interaction with HspB6. Mutations of HspB6 compromise its interaction with the WT HspB1 and significant portion of HspB6 was not included in heterooligomers. The interaction of HspB5 and HspB6. The WT HspB5 and HspB6 form definite heterooligomeric complex with stoichiometry HspB5/HspB6 close to 2:1. Mutations of HspB5 NTD weakly affect interaction with HspB6 and the stoichiometry HspB5/HspB6 in heterooligomers was larger than 2:1. Mutations of HspB6 compromise its interaction with HspB5 and different heterooligomeric complexes with variable stoichiometry were formed leaving part of HspB6 in homooligomeric state.

The formation of heterooligomeric complexes depends on many factors, such as the oligomeric state of protein partners, the accessibility of sites of interaction and, most importantly, on the presence and quantity of interaction sites. Three sites, namely the α-crystallin domain, C-terminal and N-terminal sites are involved in inter-subunit interaction. The α-Crystallin domain is present in all sHsps, and interaction through this domain is constitutive [39]. HspB1 and HspB5 have conserved IP(I/V) peptide in their C-terminal domain that is involved in inter- and intra-subunit interactions [27,31,40]. This peptide is lacking in the C-terminal domain of HspB6 and HspB8. Finally, it is supposed that the N-terminal domain takes part in formation of heterooligomeric complexes [18,24]. Oversimplifying, we can suppose that HspB1 and HspB5 have three, whereas HspB6 and HspB8 have only two potential sites involved in heterooligomerization. It was suggested that conserved (S/G)RLFD pentapeptide could interact with ACD [41] and by this means drive the interaction of different sHsps. In the case of HspB1 and HspB5, the mutation or deletion of this pentapeptide in either HspB1 or HspB5 will decrease the number of potential binding sites from six (three on HspB1 and three on HspB5) to five. As it was shown in our experiments (Figure 8), this small decrease in the number of interaction sites will not dramatically affect the heterooligomerization of these two proteins. In the case of HspB1/HspB6 or HspB5/HspB6, the WT proteins will have five potential sites of interaction (three sites on HspB1 or HspB5 and two sites on HspB6). Mutations in the N-terminal domain of HspB1 or HspB5 will decrease the number of interaction sites from five to four (two sites on HspB1 or HspB5 and two sites on HspB6). In this case, the sites of interaction are equally distributed on sHsps, forming large (HspB1 and HspB5) and small (HspB6) oligomers. Therefore, the WT HspB6 incorporates into large oligomers of HspB1 and HspB5 and forms heterooligomers with definite stoichiometry (Figure 3, Figure 5 and Figure 9). In the pairs formed by the WT HspB1 and HspB5 and mutated HspB6 the number of interaction sites will again be decreased from five to four (three sites on HspB1 and HspB5 and only one site on HspB6). In this case, the subunits of HspB1 and HspB5 tightly interact with each other and HspB6 having only ACD interaction interface will induce partial dissociation of large oligomers formed by HspB1 and HspB5 but will not be able to form heterooligomeric complexes with definite stoichiometry (Figure 4, Figure 6 and Figure 9). In our hands, the interaction of HspB8 with any sHsps is very weak, and therefore we were unable to determine the effect of mutations in the N-terminal domain of this protein on formation of heterooligomers.

At this moment, little is known about the 3D structure of heterooligomers. The point mutations, and especially the five-residue deletions within the NTD studied here, are likely to affect the contacts made by these domains. In large homooligomers of HspB5 [10] and HspB1 [42], the ACDs associate into dimers, while the NTDs are ordered to a large extent and form trimers. In the case of HspB4 [9] the ACDs still form dimers, whereas the NTDs form either dimeric or tetrameric contacts. With the structure of homooligomers in hand, it is logical to suggest that the NTDs of the two partners would be involved in the direct dimeric, trimeric or tetrameric contacts. However, contacts between the NTD of one partner and the ACD of another, like those seen in the HspB2/B3 (3:1) heterooligomers [37], are also feasible. Interestingly, the NTD mutations affecting the heterooligomer formation result in changes within this ACD interface (a possibly indirect effect) [24]. The latter observation suggests that the mechanism of mutations located in the NTD on the overall structure and properties of sHsp heterooligomers may be rather complex. Beyond artificial mutations like the ones used here, this observation is also relevant for natural, abundantly observed disease-related mutations [30,43,44].

## 4. Material and Methods

### 4.1. Cloning of the Wild-Type Proteins and Their Mutants

The cDNA of the WT HspB1, HspB5, HspB6 and HspB8 in pET22b or pET23a [45] were used for obtaining the so-called R/A mutants. Plasmids containing full-size sequences coding R/A mutants HspB1 R27A, HspB5 R22A, HspB6 R27A, HspB8 R29A were obtained in Eurogen. Plasmids containing full size sequences coding the so-called delta mutants of HspB1 Δ26–30, HspB5 Δ21–25, HspB6 Δ26–30 and HspB8 Δ28–32 were also prepared in Eurogen. All constructs were cloned into pET22b(+) or pET23a(+) vectors at *NdeI* and *XhoI* sites and integrity and lack of additional mutations were confirmed by DNA sequencing.

### 4.2. Expression and Purification of Recombinant Proteins

The expression of the WT proteins and their mutants was performed in *Escherichia coli* BL21(DE3) PLysS. For the expression of HspB6 R27A, HspB8 R29A and HspB8 Δ28–32 bacteria were grown on standard Luria-Bertani (LB) media at 37 °C. After the optical density at 600 nm reached 0.6–0.8, expression was started by addition of 0.5 mM IPTG and lasted for 6 h. Alternatively, for the production of HspB1 WT, HspB5 WT and its mutants, HspB6 WT, HspB6 Δ26–30 and HspB8 WT bacteria were grown on 3-fold LB media. In this case, bacteria at first were grown for 8 h at 37 °C followed by incubation overnight at 30 °C. 

The purification of proteins was performed by earlier described methods [30,35,45]. Briefly, in the case of HspB1 and HspB5, the proteins were subjected to ammonium sulfate fractionation followed by ion-exchange chromatography on HiTrap Q and size-exclusion chromatography on Superdex 200. In the case of HspB6 and HspB8, after ammonium sulfate fractionations, the proteins were subjected to hydrophobic chromatography on HiTrap Phenyl-Sepharose, followed by size-exclusion chromatography on Superdex 200. No less than two preparations of the WT proteins and their mutants were used for this study. The purity of proteins were nor less than 95% according to SDS gel electrophoresis [46] and no significant difference was observed for different batches of the same protein. The yield of recombinant proteins varied between 20 and 120 mg from 1 L of culture. All samples were frozen and stored at −20 °C in buffer containing 20 mM Tris-acetate pH 7.6, 10 mM NaCl, 0.1 mM EDTA, 0.1 mM phenylmethanesulphonyl fluoride (PMSF) and 2 mM dithiothreitol (DTT). The protein concentration was determined spectrophotometrically using A_280_^0.1%^ equal to 1.775 for HspB1 (UniProtKB P04792), 0.692 for HspB5 (UniProtKB P02489), 0.582 for HspB6 (UniProtKB O14558) and 1.225 for HspB8 (UniProtKB Q9UJY1).

### 4.3. Formation of Heterooligomeric Complexes

Isolated proteins were reduced by incubation in the presence of 5 mM DTT for 30 min at 37 °C. Afterwards, the proteins were pairwise mixed in buffer B (20 mM Tris-acetate, 10 mM NaCl, 150 mM NaCl, 0.1 mM EDTA, 15 mM mercaptoethanol) to obtain equimolar mixture (final concentration of each protein varied in the range of 30–40 µM). Thus, the obtained mixtures were preincubated for 1 h either at 4 °C (the rate of subunits exchange is usually negligible low) or at 42 °C (the rate of subunit exchange is high). Alternatively, equimolar mixture of analyzed proteins (30–40 µM) after reduction were mixed and incubated in buffer D (20 mM phosphate buffer pH 6.8, 0.1 mM PMSF, 15 mM mercaptoethanol) either at low (4 °C) or at high (42 °C) temperature for 1 h.

### 4.4. Analysis of Heterooligomeric Complexes

Two methods were used for the detection of the heterooligomeric complexes. In the case of sHsps with different molecular weights, i.e., in the case of HspB1/HspB6, HspB1/HspB8 and HspB5/Hsp6, the formation of heterooligomeric complexes was detected by means of size-exclusion chromatography. Samples (80–100 µL) of isolated proteins or their mixture preincubated either at low or high temperature were loaded on Superdex 200 HR 10/30 column equilibrated with buffer B. The column was run at the rate of 0.5 mL/min and 400 µL samples were collected. The proteins were precipitated by trichloroacetic acid (TCA) and after washing with acetone the protein pellet was dissolved in SDS sample buffer and subjected to SDS gel electrophoresis [46]. After staining and de-staining, the gels were scanned, and intensities of bands were evaluated by using Gel Analyzer program. The column was calibrated by using the following protein standards: thyroglobulin (669 kDa), ferritin (440 kDa), glyceraldehyde-3-phosphate dehydrogenase (144 kDa), bovine serum albumin (68 kDa), ovalbumin (43 kDa), chymotrypsinogen (25 kDa) and RNAse (13.7 kDa).

In the case of sHsps with similar large molecular weights, i.e., in the case of HspB1/HspB5, the formation of heterooligomeric complexes was determined by means of ion-exchange chromatography. Theoretically determined isoelectric points of HspB1 and HspB5 are equal to 5.98 and 6.76, respectively. Therefore, at pH 6.8, HspB1 will be tightly bound, whereas HspB5 will not be bound to the DEAE HiTrap column. Under these conditions, the heterooligomeric complexes formed by these proteins will have intermediate charge and will be weakly bound on the DEAE HiTrap column. Isolated proteins or their mixture (80–100 µL) were loaded on 1 mL DEAE HiTrap column equilibrated with buffer D (20 mM phosphate buffer pH 6.8, 0.1 mM PMSF, 15 mM mercaptoethanol). The column was washed with 5 column volumes of buffer D and eluted with 20 column volumes of linear salt gradient (0–500 mM NaCl). The column was run at the rate of 1 mL/min and 400 µL fractions were collected. Proteins were precipitated with TCA, protein pellet was washed with acetone, dissolved in SDS sample buffer and subjected to SDS gel electrophoresis. After staining and de-staining, the gels were scanned, and the intensity of each protein band was determined by the Gel Analyzer program. 

The effectiveness of heterooligomerization was analyzed using a correlation coefficient:

(1) $$\mathrm{Corr}=\frac{\sum^{\;}A_{i}B_{i}}{\sqrt{\sum^{\;}A_{i}{}^{2}}\sqrt{\sum^{\;}B_{i}{}^{2}}}$$

where *A_i_* and *B_i_* are intensities of protein bands in fraction *i.*

## Figures and Tables

**Table 1:** Correlation coefficient and molar ratio HspB1/HspB5, HspB1/HspB6 and HspB5/HspB6 in heterooligomers (in brackets).

		WT HspB1	R/A HspB1	Δ HspB1	WT HspB5	R/A HspB5	Δ HspB5
WT HspB5	Without heating	0	0	0			
	After heating	1.00 (constant ~1.0)	1.00 (constant ~1.0)	1.00 (constant ~1.0)			
R/A HspB5	Without heating	0					
	After heating	1.00 (constant ~1.0)					
Δ HspB5	Without heating	0					
	After heating	1.00 (constant ~1.0)					
WT HspB6	Without heating	0.05	**0.31 (variable 5–1)**	**0.82 (variable 3–1)**	0.05	0.12	0
	After heating	0.99 (constant ~1.1)	0.99 (constant ~1.1)	1.00 (constant ~1.2)	0.99 (constant ~2.0)	0.99 **(variable 3–2)**	0.98 **(variable 5–2)**
R/A HspB6	Without heating	0.04			0.12		
	After heating	**0.78 (variable 10–1)**			0.99 **(variable 4–1)**		
Δ HspB6	Without heating	0.05			0		
	After heating	**0.32 (variable 10–1)**			0.98 **(variable 4–1)**		
WT HspB8	Without heating	0	0	0	0	0	0
	After heating	0	0	0	0	0	0
R/A HspB8	Without heating	0			0		
	After heating	0			0		
Δ HspB8	Without heating	0			0		
	After heating	0			0		

Correlation coefficient was calculated as described in the Materials and Methods section. If the correlation coefficient was larger than 0.30, we calculated the molar ratio HspB1/HspB5, HspB1/HspB6 and HspB5/HspB6 in different fractions of size-exclusion chromatography (Figure 3, Figure 4, Figure 5, Figure 6, Figure 7 and Figure 8). Variations of this molar ratio are indicated in brackets. Values deviating from the WT case are in bold.

## Abbreviations

DTT	dithiothreitol
ME	mercaptoethanol
PMSF	phenylmethanesulphonyl fluoride
SDS	sodium dodecyl sulfate
SEC	size-exclusion chromatography
TCA	trichloroacetic acid
WT	wild type

## References

[1] Haslbeck M., Vierling E. (2015). A first line of stress defense: Small heat shock proteins and their function in protein homeostasis. J. Mol. Biol..

[2] Janowska M.K., Baughman H.E.R., Woods C.N., Klevit R.E. (2019). Mechanisms of small heat shock proteins. Cold Spring Harb. Perspect. Biol..

[3] Bakthisaran R., Tangirala R., Rao C.M. (2015). Small heat shock proteins: Role in cellular functions and pathology. Biochim. Biophys. Acta.

[4] Fontaine J.M., Rest J.S., Welsh M.J., Benndorf R. (2003). The sperm outer dense fiber protein is the 10th member of the superfamily of mammalian small stress proteins. Cell Stress Chaperones.

[5] Kappe G., Franck E., Verschuure P., Boelens W.C., Leunissen J.A., de Jong W.W. (2003). The human genome encodes 10 alpha-crystallin-related small heat shock proteins: Hspb1-10. Cell Stress Chaperones.

[6] Vos M.J., Kanon B., Kampinga H.H. (2009). Hspb7 is a sc35 speckle resident small heat shock protein. Biochim. Biophys. Acta.

[7] Taylor R.P., Benjamin I.J. (2005). Small heat shock proteins: A new classification scheme in mammals. J. Mol. Cell. Cardiol..

[8] Fang X., Bogomolovas J., Trexler C., Chen J. (2019). The bag3-dependent and -independent roles of cardiac small heat shock proteins. JCI Insight.

[9] Kaiser C.J.O., Peters C., Schmid P.W.N., Stavropoulou M., Zou J., Dahiya V., Mymrikov E.V., Rockel B., Asami S., Haslbeck M. (2019). The structure and oxidation of the eye lens chaperone alphaa-crystallin. Nat. Struct. Mol. Biol..

[10] Braun N., Zacharias M., Peschek J., Kastenmuller A., Zou J., Hanzlik M., Haslbeck M., Rappsilber J., Buchner J., Weinkauf S. (2011). Multiple molecular architectures of the eye lens chaperone alphab-crystallin elucidated by a triple hybrid approach. Proc. Natl. Acad. Sci. USA.

[11] Kriehuber T., Rattei T., Weinmaier T., Bepperling A., Haslbeck M., Buchner J. (2010). Independent evolution of the core domain and its flanking sequences in small heat shock proteins. FASEB J..

[12] Suzuki A., Sugiyama Y., Hayashi Y., Nyu-i N., Yoshida M., Nonaka I., Ishiura S., Arahata K., Ohno S. (1998). Mkbp, a novel member of the small heat shock protein family, binds and activates the myotonic dystrophy protein kinase. J. Cell Biol..

[13] Sugiyama Y., Suzuki A., Kishikawa M., Akutsu R., Hirose T., Waye M.M., Tsui S.K., Yoshida S., Ohno S. (2000). Muscle develops a specific form of small heat shock protein complex composed of mkbp/hspb2 and hspb3 during myogenic differentiation. J. Biol. Chem..

[14] Mymrikov E.V., Riedl M., Peters C., Weinkauf S., Haslbeck M., Buchner J. (2020). Regulation of small heat-shock proteins by hetero-oligomer formation. J. Biol. Chem..

[15] Zantema A., Verlaan-De Vries M., Maasdam D., Bol S., van der Eb A. (1992). Heat shock protein 27 and alpha b-crystallin can form a complex, which dissociates by heat shock. J. Biol. Chem..

[16] Kato K., Shinohara H., Goto S., Inaguma Y., Morishita R., Asano T. (1992). Copurification of small heat shock protein with alpha b crystallin from human skeletal muscle. J. Biol. Chem..

[17] Kato K., Goto S., Inaguma Y., Hasegawa K., Morishita R., Asano T. (1994). Purification and characterization of a 20-kda protein that is highly homologous to alpha b crystallin. J. Biol. Chem..

[18] Delbecq S.P., Rosenbaum J.C., Klevit R.E. (2015). A mechanism of subunit recruitment in human small heat shock protein oligomers. Biochemistry.

[19] Fontaine J.M., Sun X., Benndorf R., Welsh M.J. (2005). Interactions of hsp22 (hspb8) with hsp20, alphab-crystallin, and hspb3. Biochem. Biophys. Res. Commun..

[20] Sun X., Fontaine J.M., Rest J.S., Shelden E.A., Welsh M.J., Benndorf R. (2004). Interaction of human hsp22 (hspb8) with other small heat shock proteins. J. Biol. Chem..

[21] Bukach O.V., Seit-Nebi A.S., Marston S.B., Gusev N.B. (2004). Some properties of human small heat shock protein hsp20 (hspb6). Eur. J. Biochem..

[22] Bukach O.V., Glukhova A.E., Seit-Nebi A.S., Gusev N.B. (2009). Heterooligomeric complexes formed by human small heat shock proteins hspb1 (hsp27) and hspb6 (hsp20). Biochim. Biophys. Acta.

[23] Heirbaut M., Lermyte F., Martin E.M., Beelen S., Verschueren T., Sobott F., Strelkov S.V., Weeks S.D. (2016). The preferential heterodimerization of human small heat shock proteins hspb1 and hspb6 is dictated by the n-terminal domain. Arch. Biochem. Biophys..

[24] Heirbaut M., Lermyte F., Martin E.M., Beelen S., Sobott F., Strelkov S.V., Weeks S.D. (2017). Specific sequences in the n-terminal domain of human small heat-shock protein hspb6 dictate preferential hetero-oligomerization with the orthologue hspb1. J. Biol. Chem..

[25] Mymrikov E.V., Seit-Nebi A.S., Gusev N.B. (2012). Heterooligomeric complexes of human small heat shock proteins. Cell Stress Chaperones.

[26] Arrigo A.P. (2013). Human small heat shock proteins: Protein interactomes of homo- and hetero-oligomeric complexes: An update. FEBS Lett..

[27] Hochberg G.K., Benesch J.L. (2014). Dynamical structure of alphab-crystallin. Prog. Biophys. Mol. Biol..

[28] Hilton G.R., Lioe H., Stengel F., Baldwin A.J., Benesch J.L. (2012). Small heat-shock proteins: Paramedics of the cell. Top. Curr. Chem..

[29] Jehle S., Vollmar B.S., Bardiaux B., Dove K.K., Rajagopal P., Gonen T., Oschkinat H., Klevit R.E. (2011). N-terminal domain of alphab-crystallin provides a conformational switch for multimerization and structural heterogeneity. Proc. Natl. Acad. Sci. USA.

[30] Muranova L.K., Weeks S.D., Strelkov S.V., Gusev N.B. (2015). Characterization of mutants of human small heat shock protein hspb1 carrying replacements in the n-terminal domain and associated with hereditary motor neuron diseases. PLoS ONE.

[31] Clouser A.F., Baughman H.E., Basanta B., Guttman M., Nath A., Klevit R.E. (2019). Interplay of disordered and ordered regions of a human small heat shock protein yields an ensemble of ‘quasi-ordered’ states. eLife.

[32] Peschek J., Braun N., Rohrberg J., Back K.C., Kriehuber T., Kastenmuller A., Weinkauf S., Buchner J. (2013). Regulated structural transitions unleash the chaperone activity of alphab-crystallin. Proc. Natl. Acad. Sci. USA.

[33] Heirbaut M., Beelen S., Strelkov S.V., Weeks S.D. (2014). Dissecting the functional role of the n-terminal domain of the human small heat shock protein hspb6. PLoS ONE.

[34] Weeks S.D., Muranova L.K., Heirbaut M., Beelen S., Strelkov S.V., Gusev N.B. (2018). Characterization of human small heat shock protein hspb1 alpha-crystallin domain localized mutants associated with hereditary motor neuron diseases. Sci. Rep..

[35] Shatov V.M., Weeks S.D., Strelkov S.V., Gusev N.B. (2018). The role of the arginine in the conserved n-terminal domain rlfdqxfg motif of human small heat shock proteins hspb1, hspb4, hspb5, hspb6, and hspb8. Int. J. Mol. Sci..

[36] Rogalla T., Ehrnsperger M., Preville X., Kotlyarov A., Lutsch G., Ducasse C., Paul C., Wieske M., Arrigo A.P., Buchner J. (1999). Regulation of hsp27 oligomerization, chaperone function, and protective activity against oxidative stress/tumor necrosis factor alpha by phosphorylation. J. Biol. Chem..

[37] Clark A.R., Vree Egberts W., Kondrat F.D.L., Hilton G.R., Ray N.J., Cole A.R., Carver J.A., Benesch J.L.P., Keep N.H., Boelens W.C. (2018). Terminal regions confer plasticity to the tetrameric assembly of human hspb2 and hspb3. J. Mol. Biol..

[38] Horwitz J. (2003). Alpha-crystallin. Exp. Eye Res..

[39] Clark A.R., Lubsen N.H., Slingsby C. (2012). Shsp in the eye lens: Crystallin mutations, cataract and proteostasis. Int. J. Biochem. Cell Biol..

[40] Delbecq S.P., Jehle S., Klevit R. (2012). Binding determinants of the small heat shock protein, alphab-crystallin: Recognition of the ‘ixi’ motif. EMBO J..

[41] Klevit R.E. (2020). Peeking from behind the veil of enigma: Emerging insights on small heat shock protein structure and function. Cell Stress Chaperones.

[42] Nappi L., Aguda A.H., Nakouzi N.A., Lelj-Garolla B., Beraldi E., Lallous N., Thi M., Moore S., Fazli L., Battsogt D. (2020). Ivermectin inhibits hsp27 and potentiates efficacy of oncogene targeting in tumor models. J. Clin. Investig..

[43] Datskevich P.N., Nefedova V.V., Sudnitsyna M.V., Gusev N.B. (2012). Mutations of small heat shock proteins and human congenital diseases. Biochemistry.

[44] Muranova L.K., Ryzhavskaya A.S., Sudnitsyna M.V., Shatov V.M., Gusev N.B. (2019). Small heat shock proteins and human neurodegenerative diseases. Biochemistry.

[45] Mymrikov E.V., Bukach O.V., Seit-Nebi A.S., Gusev N.B. (2010). The pivotal role of the beta 7 strand in the intersubunit contacts of different human small heat shock proteins. Cell Stress Chaperones.

[46] Laemmli U.K. (1970). Cleavage of structural proteins during the assembly of the head of bacteriophage t4. Nature.

